# Cardiothoracic CTA in Infants Referred for Aortic Arch Evaluation—Retrospective Comparison of Iomeprol 350, Ioversol 350, Iopromide 370 and Iodixanol 320

**DOI:** 10.3390/children8110949

**Published:** 2021-10-21

**Authors:** Marian Pop

**Affiliations:** 1 ME1 Department, “George Emil Palade” University of Medicine Pharmacy Sciences and Technology of Tirgu Mures, 540142 Tirgu Mures, Romania; marian.pop@umfst.ro; Tel.: +40-749-260-920; 2 Radiology and Medical Imaging Department, Tirgu Mures Emergency Institute for Cardiovascular Diseases and Heart Transplant, 540136 Tirgu Mures, Romania

**Keywords:** contrast agents, computed tomography angiography(CTA), congenital cardiovascular malformations, aortic arch

## Abstract

Background: Computed tomography angiography (CTA) in infants is considered one of the most challenging radiological examinations due to difficulties in balancing start delay, contrast agent (CA) volume and flow in order to achieve optimal opacification of the large vessels. This study aimed to compare the contrast enhancement achieved by four CAs when taking into consideration CA injection parameters and patient characteristics. Methods: We performed a retrospective assessment of forty-eight consecutive cardiothoracic CTAs performed for aortic arch evaluation on children aged 0–1 year. All examinations were performed using the same 64-slice scanner and power injector using the bolus tracking technique. Axial 0.6 mm slices were used to measure large vessel enhancement using regions of interest at the level of the main pulmonary artery, ascending and descending aorta. The recorded variables included anthropometric measurements, CA type, flow rate, volume, and the average Hounsfield unit (HU) values of the blood pool. Descriptive statistics are presented as averages and standard deviations (SD) for normal distributed data or otherwise as medians and interquartile ranges (IQRs). Results: We found no statistically significant differences between age and anthropometric parameters when looking at different CAs. The median CA volume was 7 (IQR, 7–9) mL with the average flow rate of 0.94 (SD, 0.23) mL/s. Ascending aorta average HU values were 605.9 (SD, 177.23) for Iomeprol 350, 626 (SD, 183.83) for Ioversol 350, 530.83 (SD, 175.56) for Iopromide 370 and 354.91 (SD, 115.81) for Iodixanol 320. The difference in HU value for Iodixanol 320 compared to the other CAs was statistically significant. Similar differences were found for the other vascular structures. Conclusion: In CTA of infants suspected of aortic arch hypoplasia/coarctation, Iodixanol 320 provided up to 40% less enhancement of the great vessels when compared to Iomeprol 350, Ioversol 350 and Iopromide 370.

## 1. Introduction

Approximately 1% of born children will be affected by congenital heart disease [1]; therefore, it is essential to maximize the safety and efficacy of diagnostic procedures.

Advances in computed tomography (CT) technology have extended its application in the imaging of pediatric patients with congenital heart disease [2], with computed tomography angiography (CTA) playing a particularly important role in the evaluation of both thoracic and vascular malformations in young children [3].

CTA is being used more and more, with indications well documented [4,5] and good results [6,7], but its diagnostic precision in children is dependent on optimal image quality [8].

Good images are obtained by having the maximal concentration of contrast in the vessel of importance at the time of acquisition [9] with technical challenges including the small volumes, the slow injection rate of the contrast agent (CA) and the use of small gauge canula located in tiny peripheral veins [10]. Moreover, existing reports [11] suggested that the type of contrast may affect the enhancement of vascular structures.

Depending on the chemical structure the CA can be classified as ionic/non-ionic and monomer/dimer with different properties, clinical uses and toxicity profiles [12]. Different types of iodine-based contrast agents are available for CTA, with different osmolarities and viscosity and osmolarity [13].

In adults and phantoms, previous studies have compared CTA vascular enhancement when using 2 [14,15,16,17,18,19,20], 4 [21] or 5 [22,23] contrast agents. Even if the mean enhancement was lower in iso-osmolar CA [21,24], it provided good-quality images.

The purpose of this study was to perform a retrospective assessment of contrast enhancement for several large thoracic vessels in infants referred for aortic arch evaluation, considering the CA volume, flow, iodine flow rate, patient anthropometric and demographic parameters.

This study is the first to investigate the CTA contrast enhancement of large thoracic vessels in infants referred for evaluation of aortic arch when using one of the four different CAs: Iomeprol 350, Ioversol 350, Iopromide 370 and Iodixanol 320.

## 2. Materials and Methods

### 2.1. Study Population and CTA Data

The study population comprised 205 consecutive pediatric cardiothoracic CTA examinations performed between 2015 and 2020.

The inclusion criteria were age under 365 days and referral for CTA of the aortic arch; the exclusion criteria were represented by unfinished CTA, which, in the end, yielded a sample size of 48.

All examinations were performed using a 64-slice multidetector CT (MDCT) scanner (Definition AS, Siemens, Erlangen, Germany). Per local acquisition protocols, ECG gating, a tube voltage of 70 to 100 kV and automated tube current modulation was used. A single-phase arterial bolus was planned using bolus detection with ROI in the descending aorta and a threshold of 100 HU. The CAs (Iomeprol 350, Ioversol 350, Iopromide 370 or Iodixanol 320) were administered using a power injector (Mallinckrodt OptiVantage, Cincinnati, OH, USA) with flows adapted to the body habitus (0.5–1.6 mL/s), no saline flush and no contrast dilution. The total contrast volume ranged from 4 to 15 mL.

**Figure 1:**
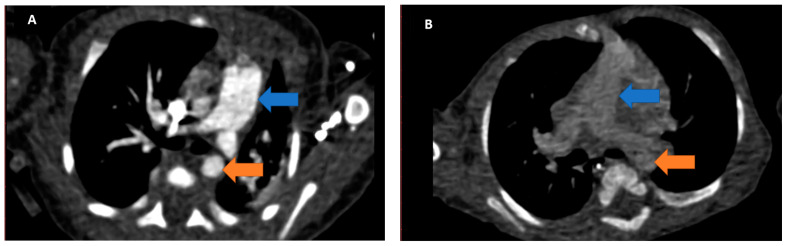
Axial CT angiography reconstruction. Blue arrow: main pulmonary artery. Orange arrow: descending aorta. (**A**) Excellent quality of image. The vascular structures are well-delineated due to good contrast opacification; (**B**) Suboptimal image quality. The vessels are difficult to delineate and reporting is impaired.

Images were reconstructed using a 0.6 mm slice thickness with a soft kernel (B26f) and a field of view encompassing the thorax (see Figure 1 for sample).

A radiologist with more than 8 years of experience in cardiovascular imaging, blinded to the type of CA used, measured the average blood pool HU values in the ascending aorta, main pulmonary artery and descending aorta using round regions of interest covering the lumen of the respective corresponding vessels (Medixant, RadiAnt DICOM Viewer ellipse tool).

The study was performed in accordance with the 1964 Declaration of Helsinki ethical standards and was approved by the institutional review board of the Emergency Institute for Cardiovascular Diseases and Heart Transplant. Informed consent was obtained from the parents/legally authorized representatives of the participants before CTA examination.

### 2.2. Statistical Analysis

Data analysis was performed using SPSS [25]. Continuous variables were tested for normal distribution using the D’Agostino–Pearson test. Normally distributed continuous variables were presented as means (standard deviation (SD)), while non-normally distributed variables were presented as medians (interquartile range (IQR)). Categorical variables were reported as count and percentage.

A one-way analysis of variance (ANOVA) or Kruskal–Wallis test was used to test for statistical significance between groups. A two-sided *p* value of <0.05 was considered significant.

## 3. Results

### 3.1. Patient Population

Of the 48 participants, 23 (47.9%) were male. The median age of the sample was 10.5 (IQR, 6–74.5) days, and median weight was 3.37 (IQR, 2.9–4) kg (Table 1). The scanning was performed in 89.5% of the cases using 80 kV.

Subgroup analysis based on the CA type found no statistically significant differences between median age (*p* = 0.056), weight (*p* = 0.58) and length (*p* = 0.33).

### 3.2. Contrast Agent Data

The median CA volume was 7 (IQR, 7–9) mL, and the average flow rate was 0.94 (SD, 0.23) mL/s.

When looking at subgroups, as defined by the type of CA being used, we found no statistically significant differences when looking at the volume or iodine injection flow (defined as mg of iodine/kg of body weight/s), while the average flow in the case of Iodixanol 320 was found to be significantly higher (*p* = 0.03). Nevertheless, the average iodine dosage (defined as mg of iodine/ kg of body weight) was significantly lower in the case of Iodixanol 320 (*p* < 0.001), with an average value of 554.81 mg I/kg compared with Iomeprol 350 (816.66 mg I/kg), Ioversol 350 (820.56 mg I/kg) and Iopromide 370 (795.75 mg I/kg) (Table 2).

### 3.3. Thoracic Vessel Enhancement Comparison

**Figure 2:**
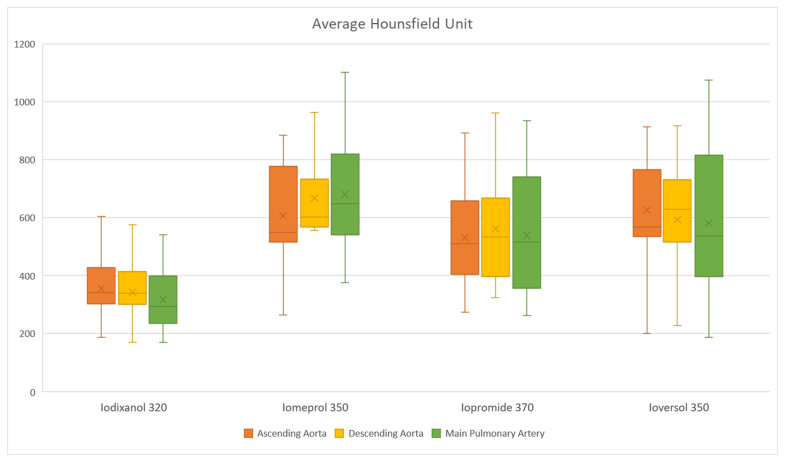
Comparison of the average HU in the ascending aorta, descending aorta and main pulmonary artery, grouped by the contrast agent used.

Average blood pool HU was lowest in CTA examinations using Iodixanol 320 (Figure 2), with a statistically significant difference between Iodixanol 320 and all other CA types. The HU values for Iodixanol 320 were 60% of the total average HU value at the main pulmonary artery (316.66 compared to 526.66), 66.98% at the ascending aorta (354.91 compared to 529.83), and 62.96% at the descending aorta (345.5 compared to 548.75) (Table 3).

## 4. Discussion

This study compared the contrast enhancement of four CAs used in CTA in infants with suspected aortic arch hypoplasia/coarctation. Our primary finding was that for patients of a similar age and size, when using a similar contrast volume and flow rate, Iodixanol 320 provided up to 40% less enhancement of the vascular structures than Iomeprol 350, Ioversol 350 and Iopromide 370.

While our results indicate that CA affects large vessels’ contrast enhancement, optimal CTA imaging is also reliant on multiple factors related to machine parameters and technique [3].

In our study, the average injection flow rate was adjusted for the infants, taking into consideration the IV gauge and IV site [5,10] in order to limit the volume of CA injected into the recommended CA volume used in pediatric CTA of 1–2 mL/kg [4,5,10]. The flow ranged overall from 0.5 to 1.6 mL/s, being highest in the Iodixanol group (1.08 mL/s) but being similar to flows reported in the literature [2,3,26].

The bolus detection technique is well documented, [22] and previous studies have suggested that using the bolus detection technique with a consistent acquisition protocol should yield similar enhancement despite differing CAs in normally developing infants [27] with properly assessed [28] nutrition.

Using low-kV to increase contrast enhancement is an established technique [29,30], and 80kV was used in 90% of our patients. However, 5 of our patients had been scanned with different kV (four from the Iodixanol 320 group being scanned at 70 kV and one from the Iopromide 370 group at 100 kV).

Most of the studies comparing different CA for CTA have evaluated adult patients. In such studies, it was shown that Iodixanol 320 can provide vascular enhancement that is comparable to Iohexol 350 [14,17]. However, there is no consensus since Honoris [21] found that even in adults vascular enhancement across all coronary segments was highest in Iopamidol followed by Iohexol, iodixanol 320 and iodixanol 270, respectively (*p* < 0.002). Still, such studies have been using a higher flow rate of 4 [14] or 5 mL/s [17,21] and a much larger volume of contrast. Reports also exist about Iodixanol providing a higher enhancement than Iomeprol 400 [31], but this occurred when the iodine delivery rate was identical, with volumes and flow rate adjusted for the adult population (4–5 mL/s).

With regard to pediatric patients, Hwang [26] compared Iodixanol 270 with Ioptomide 370 and found no statistically significant difference between Iodixanol 270 and Iopromide 370. While they used a faster dual-source machine and heated CA, it must be noted that Iodixanol 270 has half the viscosity of Iodixanol 320 [32].

Taking into account the viscosity, experimental studies [23] showed that the highest iodine delivery rates across different catheter gauges were for Iopromide, followed by Iohexol 350, Iomeprol 400 and then Iodixanol 320. The effect of CA viscosity [23] on contrast enhancement might be compensated by higher iodine concentration, as is the case for the average HU values in the ascending aorta, where we have an non-significant difference between Iopromide 370 (530 HU) and Ioversol 350 (626 HU) but a significant one between Iodixanol 320 (354 UH) and any other assessed CA.

We postulate that our results are due to differences in iodine concentration as well as differences in the intrinsic flow and vascular distribution of CAs in this population, as Iodixanol 320, the CA with the lowest measured enhancement, has the highest viscosity at room temperature [33]. While we stopped using Iodixanol 320 for infants’ CTA, we acknowledge the fact that newer CT machines and other injection protocols may yield different results; however, for the time being, our experience shows a certain advantage in using CAs with a higher iodine concentration.

### Limitations

Several limitations of our study warrant mentioning. First, this was a single-center, small-sample observational study. However, as a comparison study, its design is useful and draws valid conclusions. Second, the CTA examination was performed using a 64-slice scanner; however, newer machines with larger detectors and faster acquisition times could use acquisition protocols that, with similar CA injection parameters, may yield different results.

Finally, a limitation lies within the small number of participants. Because the conditions being evaluated (aortic arch hypoplasia/coarctation) are so rare, the number of CTA required for aortic arch evaluation is also small. However, also because these examinations are rare, the findings of this study offer new, potentially useful information for this patient population.

## 5. Conclusions

In CTA of infants suspected of aortic arch hypoplasia/coarctation, Iodixanol 320 achieved up to 40% less enhancement of the great vessels when compared to Iomeprol 350, Ioversol 350 and Iopromide 370.

## Figures and Tables

**Table 1:** Patient characteristics and tube voltage.

	Total	Iomeprol 350	Ioversol 350	Iopromide 370	Iodixanol 320	*p*-Value
*N*(%)	48 (100%)	11 (22.91%)	13 (27.08%)	12 (25%)	12 (25%)	
Male:Female ratio	0.92	0.57	2.25	0.71	0.71	
Age (days), Median (IQR)	10.5 (6–74)	7 (4–28)	11 (5–23)	10 (7.75–21.75)	111 (20.5–198.5)	0.056
Weight (kg), Median (IQR)	3.37 (2.9–4)	3.3 (2.95–3.72)	3.4 (2.7–4)	3.27 (2.92–3.41)	3.9 (3.17–7.11)	0.58
Length (cm), Median (IQR)	53 (51–57)	53 (51–54.5)	52 (51–55)	53 (50.75–56.5)	56.5 (52.75–67.25)	0.33
Scanned at 70 kV	4 (8.33%)				4 (100%)	
Scanned at 80 kV	43 (89.58%)	11 (25.58%)	13 (30.23%)	11 (25.58%)	8 (18.6%)	
Scanned at 100 kV	1 (2.08%)			1 (100%)		

(IQR) indicates interquartile range.

**Table 2:** Contrast agents data-related parameters.

Variables	Iomeprol 350	Ioversol 350	Iopromide 370	Iodixanol 320	*p*-Value
Volume, Median (IQR)	8 (7–8.75)	7 (6.75–9.25)	7 (6.5–8)	7 (5.5–10)	0.68
Flow, Mean (SD)	0.92 (0.17)	0.94 (0.15)	0.81 (0.15)	1.08 (0.34)	0.03 *^,†^
Iodine mg/kg body weight, Mean (SD)	816.66 (98.08)	820.56 (85.22)	795.75 (157.55)	564.81 (92.13)	<0.001 *^,†^
Iodine mg/kg body weight/s, Median (IQR)	93.33 (79.72–97.22)	96.25 (89.66–106.94)	87.13 (74.42–98.28)	77.19 (54.82–109.53)	0.33

(IQR) indicates interquartile range. SD indicates standard deviation. * Denotes statistically significant difference. ^†^ Denotes ANOVA; otherwise Kruskall–Wallis.

**Table 3:** HU average values of the large vessels.

Location	Iomeprol 350	Ioversol 350	Iopromide 370	Iodixanol 320	*p*-Value
Main pulmonary artery, Mean (SD)	679.54 (191.29)	580.69 (256.73)	538 (225.93)	316.66 (105.63)	0.001 *
Ascending aorta, Mean (SD)	605.9 (177.23)	626 (183.83)	530.83 (175.65)	354.91 (115.81)	0.001 *
Descending aorta, Mean (SD)	677 (145.68)	613.69 (190.33)	564.08 (219)	345.5 (93.96)	<0.001 *

SD indicates standard deviation. * Denotes statistically significant difference (ANOVA).

## Data Availability

The data presented in this study are available on request from the corresponding author. The data are not publicly available due to ongoing research.
